# Efficacy of Crohn’s disease exclusion diet in treatment -naïve children and children progressed on biological therapy: a retrospective chart review

**DOI:** 10.1186/s12876-023-02857-6

**Published:** 2023-06-29

**Authors:** María Clara Jijón Andrade, Gemma Pujol Muncunill, Ana Lozano Ruf, Laura Álvarez Carnero, Victor Vila Miravet, Dolores García Arenas, Natalia Egea Castillo, Javier Martín de Carpi

**Affiliations:** Unit for the Comprehensive Care of Paediatric IBD, Department of Paediatric Gastroenterology, Hepatology and Nutrition, Sant Joan de Déu Hospital, Barcelona, Spain

**Keywords:** Crohn’s disease, Crohn’s disease exclusion diet, Partial enteral nutrition, Remission, Paediatrics

## Abstract

**Background:**

Recent trials suggested that the Crohn’s disease (CD) exclusion diet (CDED) plus partial enteral nutrition (PEN) is a safe and effective strategy in remission induction of paediatric-onset CD. However, real-world evidence regarding the safety and efficacy of the CDED plus PEN approach is still lacking. The present case-series study reported our experience with the outcomes of CDED plus PEN in the paediatric-onset CD at disease onset and after the loss of response to biologics.

**Methods:**

We conducted a retrospective chart review on children who were treated with CDED plus PEN through the period from July 2019 and December 2020. Clinical and laboratory data were retrieved and compared at baseline, 6, 12, and 24 weeks of treatment. The primary endpoint of the present study was the rate of clinical remission.

**Results:**

The present study retrieved the data from 15 patients. Of them, nine patients were treatment naïve at the time of initiation of CDED plus PEN (group A) and the remaining patients relapsed on biologics before treatment. All patients in groups A and B exhibited clinical remission in week six, which was sustained until week 12. At the end of the follow-up, the clinical remission rate was 87% and 60% in groups A and B, respectively. No side effects were observed in both groups. In group A, the faecal calprotectin (FC) and albumin improved at week six, week 12, and week 24 (p < 0.05). The erythrocyte sedimentation rate (ESR) improved significantly at week 12 (p = 0.021) and week 24 (p = 0.027). At the same time, the haemoglobin and iron levels showed significant improvement only at week 24. For group B, only FC showed numerical reductions over time that did not reach the level of statistical significance.

**Conclusion:**

Treatment with CDED plus PEN was well tolerated and achieved an excellent clinical remission rate in treatment-naive patients. However, the benefit of CDED plus PEN was less in patients who initiated the strategy after losing the response to biologics.

## Background

The incidence of paediatric-onset inflammatory bowel disease (IBD) has shown a notable increase over the past few decades, approaching a 133% increase in some cohorts [1–4]. Recent reports demonstrated that the incidence of paediatric-onset IBD in Europe ranged from 0 to 21.3 per 100,000 person-years [5]. Crohn’s disease (CD) is the most common form of IBD in children and is characterised by relapsing, chronic, transmural inflammation of the digestive tract [6]. The onset of CD occurs during childhood or adolescence in nearly 25% of the cases and continues to adulthood. While the exact aetiology has not been fully elucidated yet, it is suggested that CD is a multifactorial disorder with underlying immunologic, environmental, and microbiologic factors predisposing to the immune response in CD [7]. Previous natural history studies suggested that paediatric-onset CD has a more severe presentation and aggressive course than adult-onset disease, presenting additional complexity in clinical management [7, 8]. CD can exert a substantial burden on the affected children and their families in the form of hospitalisation due to progression and severe symptoms, growth failure, impaired daily activities and quality of life (QoL), and psychological disorders [9, 10]. Besides, the economic burden of paediatric-onset CD is considerable, involving both direct medical, such as treatment and hospitalisation costs, and indirect costs, such as days missed from schools and loss of productivity of the caregivers [11, 12].

Therefore, achieving a symptoms-free status has long been considered the main treatment goal for children with CD. In recent years, novel biologics have considerably improved paediatric CD outcomes and prognosis, which changed the disease management landscape [10]. Currently, the treatment goals of paediatric-onset CD encompass maintaining the QoL and symptoms-free status, alongside the restoration of growth. Still, several therapeutic challenges are often faced, especially the loss of response to biological treatment and the considerable risk of treatment-related complications [13]. Exclusive enteral nutrition (EEN) remains an effective first-line therapy for remission induction in mild and moderate paediatric CD. Experimental evidence suggested that high animal protein and fat induce luminal inflammation in CD models [14]. However, EEN -defined as the oral administration of liquid formula via an enteral tube as the only source of caloric requirements for up to eight weeks - is a highly restrictive approach that can be associated with limited adherence, and it is not suitable for long-term courses [7, 8]. Besides, the rate of sustained clinical remission in patients receiving ENN is low, with nearly 70% of patients relapse within 12 months after treatment [15–17].

A growing number of randomised trials suggested that the CD exclusion diet (CDED) plus partial enteral nutrition (PEN) is a safe and effective strategy in remission induction of paediatric-onset CD. CDED refers to whole foods diet in combination with parenteral nutrition (PEN) that aims to minimize the intake of dietary elements that can negatively impact the microbiome (dysbiosis), intestinal barrier, and intestinal immunity [18]. CDED is hypothesized to increase the effectiveness of PEN while increasing patient compliance. Studies showed that this strategy was well tolerated and achieved remission in 80–85% of mild-moderate CD in children [18, 19]. Further studies found that the CDED plus PEN achieved promising results in other clinical scenarios, such as after the loss of response to biological therapy [13, 20–22]. Evidence from adult studies demonstrated clinical improvement and remission rates of 90% and 62%, respectively [13].

However, real-world evidence regarding the safety and efficacy of the CDED plus PEN approach is still lacking; real-world evidence can demonstrate the clinical outcomes of CDED and its role in different clinical scenarios. In our practice, the CDED plus PEN approach has been used in paediatric-onset CD since 2019. The present case-series study reported our experience with the outcomes of CDED plus PEN in the paediatric-onset CD at disease onset and after the loss of response to biologics.

## Methods

The present study was reported in concordance with the recommendation of the STROBE guideline [23].

### Study design and patients

We conducted a retrospective chart review on children (aged ≤ 17 years old) who were treated with CDED plus PEN at the Sant Joan de Déu Hospital in Barcelona, Spain, through the period from July 2019 and December 2020. Patients were included if they had documented diagnosis of paediatric-onset CD according to the Porto criteria [15] and a mild-to-moderate disease activity (defined as Paediatric Crohn’s Disease Activity Index [PCDAI] 12.5–47.5 points) at the beginning of CDED plus PEN. We retrieved the data of patients with a minimum follow-up duration of three months. Patients were excluded if they were being followed up in a different hospital, receiving EEN, or having an active perianal disease. Besides, we excluded patients who rejected CDED plus PEN on the first week of treatment. As per our institution’s guidelines, pharmacological therapy modifications were possible during the CDED plus PEN.

### Treatment and data collection

In our practice, paediatric patients with mild-to-moderate CD received CDED plus PEN according to the recommendations of the Modulife™ expert training program on CDED plus PEN (modulifexpert.com). In this program, the strategy consists of three phases with decreasing amounts of PEN based on Modulen IBD® (Nestle Health Science) polymeric formula and increasing amounts and variety of CDED [24]. Dietary support, anthropometric evaluation and clinical interviews were scheduled by a dietitian, paediatric gastroenterologist, and a specialised nurse with the role of a case manager.

The demographics (gender, age at diagnosis and age at CDED initiation) were collected from the records of eligible patients. Besides, we collected the following data at baseline, 6, 12, and 24 weeks of treatment: PCDAI, weight Z-score, faecal calprotectin (FC), haemoglobin, albumin, C-reactive protein (CRP), iron, and erythrocyte sedimentation rate (ESR). The primary endpoint of the present study was the rate of clinical remission at 6, 12 and 24 weeks of treatment, defined as PCDAI < 10 points. The secondary endpoints included adherence rate, safety outcomes, and changes in growth parameters and markers of inflammation over the 24 weeks. The adherence was evaluated during clinical interviews according to patients’ and parents’ impressions.

### Statistical analysis

The statistical analysis was conducted using IBM SPSS Statistics for Windows, Version 26.0 (BM Corp., Armonk, N.Y., USA). Summary statistics were utilised for data presentation, and categorical variables were compared with Chi^2^ and Fisher’s tests. Nonparametric continuous variables were compared using the Mann-Whitney U test. A p-value of less than 0.05 was considered statistically significant.

## Results

The present study retrieved the data from 15 patients. Of them, nine patients were treatment naïve at the time of initiation of CDED plus PEN (group A) and the remaining patients relapsed on biologics before treatment (median time from the start of biologic treatment to loss of response was 375.5 [interquartile range [IQR] 65.8-799.3] days). Baseline characteristics are summarised in Table 1. The median baseline PCDAI was 15 (IQR 11.25–17.5) and 20 (IQR 3.75–39.38) points in groups A and B, respectively. All patients in group A received azathioprine, compared to two patients (33.3%) in group B.

**Table 1:** Group characteristics at baseline

Group A (n 9)	Group B (n 6)
Male 5, Female 4	Male 3, Female 3
Age at CD diagnosis: Median 11.8 years (IQR 10.7–14.9)	Age at CD diagnosis: Median 13.5 years (IQR 9.1–15.4)
Age at the beginning of CDED + PEN: Median 15.8 years (IQR 12.6–18.1)	Age at the beginning of CDED + PEN: Median 12 years (IQR 10.7–15)
All patients received Azathioprine	2 patients received Azathioprine 3 Adalimumab and 3 Ustekinumab
Median PCDAI: 15 points (IQR 11.25–17.5)	Median PCDAI: 20 points (IQR 3.75–39.38)
Median Calprotectin 922 mg/kg (IQR 653-1765.5)	Median Calprotectin 5248.5 mg/kg (IQR 800.75-6225.75)
Median ERS 11 mm/h (IQR 3–21)	Median ERS 8.5 mm/h (IQR 5-14.25)
Median CRP 3 mg/l (IQR 0.45–15.95)	Median CRP 15 mg/dl (IQR 1.98–22.65)
Median Albumin 40 g/dl (IQR 38–42)	Median Albumin 39.5 g/dl (IQR 33.75-44)
Median Hb 12.4 g/dl (IQR 11.9–13.3)	Median Hb 12.5 g/dl (IQR 11.87–14.1)
Median Iron 8.8 umol/l (IQR 5.7-15.65)	Median Iron 9 umol/l (IQR 5.8–11.6)

Abbreviations: number (n), interquartile range (IQR), Crohn’s disease (CD), Paediatric Crohn’s Disease Activity Index (PCDAI)

**Fig. 1:**
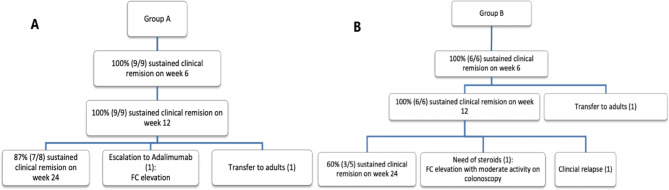
Clinical remission and outcomes in (**a**) group A and (**b**) group B

All patients in groups A and B exhibited clinical remission in week six, which was sustained until week 12. At the end of the follow-up, two patients in groups A and B were not assessed as the patient turned 18 years old and was transitioned to adult care. Seven of the remaining eight patients in group A had sustained clinical remission (87%), and one required a step-up to adalimumab due to sustained FC elevation (Fig. 1a). On the other hand, out of the five remaining patients in group B at week 24, three patients had sustained clinical remission (60%), one patient required steroids due to a relapse based on moderate activity assessed by colonoscopy and analytical parameters, and the last patient had clinical relapse requiring treatment change to ustekinumab (Fig. 1b).

**Fig. 2:**
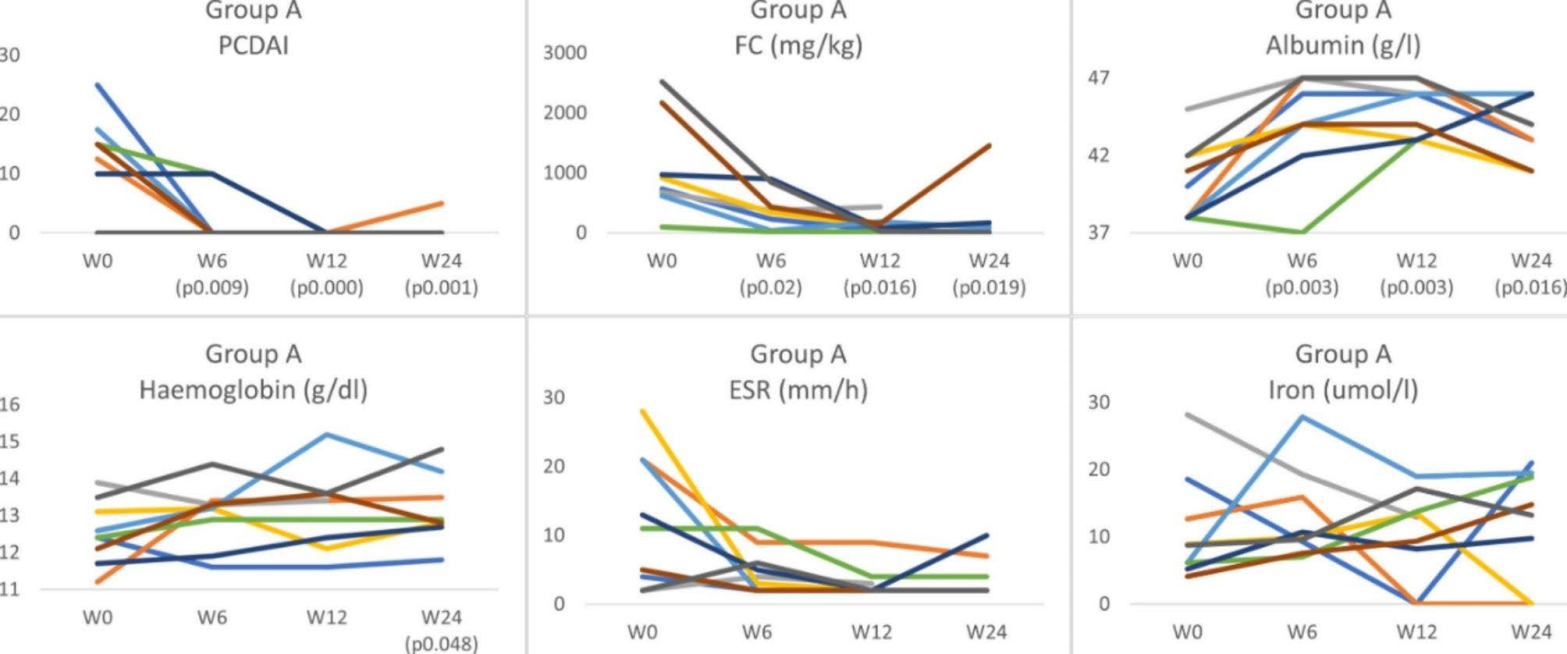
The change in PCDAI, FC, albumin, haemoglobin, ESR, and iron in group A at weeks 6, 12, and 24. PCDAI: Paediatric Crohn’s Disease Activity Index; FC: Faecal calprotectin (FC); ESR: erythrocyte sedimentation rate

**Fig. 3:**
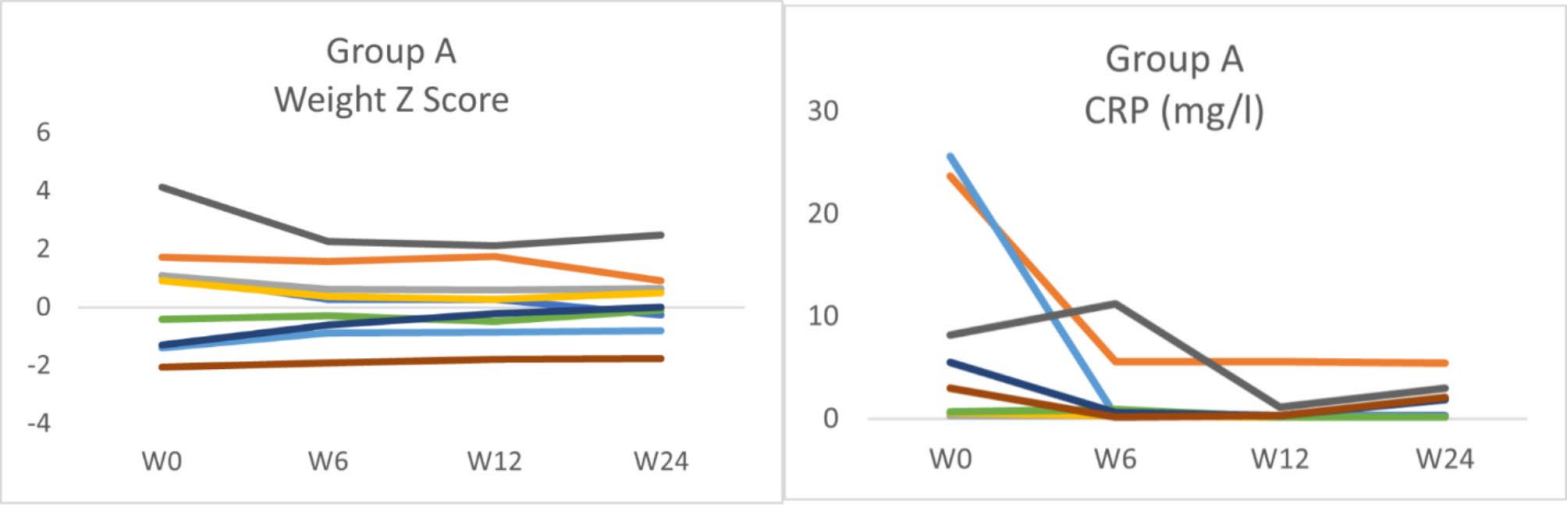
The change in Weight Z-score and CRP in group A at weeks 6, 12, and 24. CRP: C-reactive protein

**Fig. 4:**
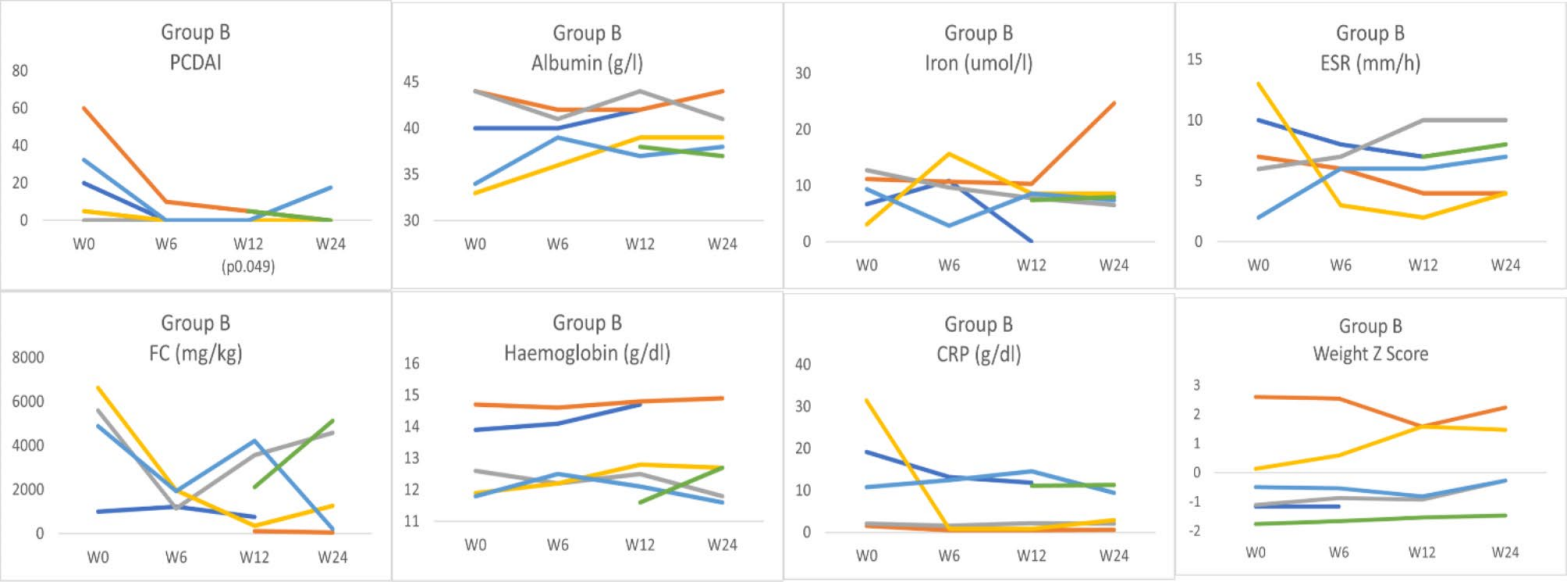
The change in PCDAI, albumin, iron, ESR, FC, haemoglobin, CRP, and weight z-score in group B at weeks 6, 12, and 24 PCDAI: Paediatric Crohn’s Disease Activity Index; FC: Faecal calprotectin (FC); ESR: erythrocyte sedimentation rate; CRP: C-reactive protein

In group A, the median reduction in PCDAI at week 6 was 13.8 points (p = 0.09), at week 12 was 15 points (p = 0.002) and at week 24 was 15 points (p = 0.006). Likewise, the FC and albumin also improved at week 6 (median reduction 585 mg/kg, p = 0.02; 4 g/L, p = 0.03, respectively), week 12 (median reduction 801 mg/kg, p = 0.016; and 5 g/l, p = 0.03, respectively) and week 24 (median reduction 723 mg/kg, p = 0.019; and 3 g/l, p = 0.016, respectively). The ESR improved significantly at week 12 (median reduction 7 mm/h, p = 0.021) and week 24 (median reduction 5 mm/h, p = 0.027). At the same time, the haemoglobin and iron levels showed significant improvement only at week 24 (median reduction 0.9 g/dl, p = 0.048; and 7.1umol/l, p = 0.003, respectively), Fig. 2. On the other hand, the CRP and weight Z score did not show significant changes throughout the follow-up period (Fig. 3). For group B, only PCDAI and FC showed numerical reductions over time that did not reach the level of statistical significance. The remaining variables did not show significant improvement (Fig. 4).

Regarding adherence, all patients in group A adhered to treatment until week 12. The same finding was observed for the eight patients who remained in paediatric care at week 24. (Table 2). The adherence rate in group B was 100% (6/6) on week 6. Before week 12, one patient had left the study (< 18 years of age), and the rest (5/5) maintained adherence on weeks 12 and week 24. No adverse events were reported in either group (Table 2).

**Table 2:** Descriptive analysis of adherence and Safety

Group A (n 9)	Group B (n 6)
Adherence: - 9/9 on week 6 - 9/9 on week 12 - 8/8 on week 24	Adherence: - 6/6 on week 6 - 5/5 on week 12 - 5/5 on week 24
- 1 was transferred to the adult unit before week 24	- 1 was transferred to the adult unit before week 12
No side effects reported	No side effects reported

number (n)

## Discussion

Dietetic treatment still represents an effective strategy for remission induction in paediatric with CD. CDED can potentially improve treatment adherence in the paediatric population, with an equal efficacy profile to EEN and a well-tolerable safety profile [25]. It is proposed that CDED positively modify intestinal microbiota by reducing pro-inflammatory bacteria, such as Proteobacteria, with subsequent reduction in the luminal inflammation, improvement in the mucosal, and restoration of adequate intestinal permeability [26–28]. However, real-world evidence regarding the effectiveness of CDED plus PEN in paediatric CD is scarce. This report presented the outcomes of CDED plus PEN in paediatric CD in different clinical scenarios.

In newly-diagnosed patients with mild-to-moderate disease activity, we found that CDED plus PEN led to an excellent clinical response at disease onset, achieving clinical remission by week six and maintaining it by week 24 in up to 87% of the patients. These results are even better than those reported with CDED plus PEN (80%) and with EEN (73%) in previous paediatric literature [8, 18, 19, 29]. The improvement of other clinical and analytical parameters, such as a decrease in PCDAI and FC and an increase in albumin throughout the entire follow-up period, supports this clinical improvement. These findings support the initial 12-week improvement in other paediatric series but further demonstrate the persistence of clinical remission over a longer follow-up period of 24 weeks [18, 19]. It is worth noting that there were no significant changes in the weight z scores and CRP level over the follow-up period. This can be explained by the fact that all patients, except one, had normal weight z scores at baseline, probably due to the nutritional support received before CDED plus PEN. The underweight patient at baseline achieved normal weight by week six and continued to improve it by week 24. Regarding the CRP, there was a notable decrease in the CRP values over the follow-up period; the lack of statistical significance could be explained by the limited sample size and the relatively low median CRPs at baseline. The prolonged efficacy of treatment over time is an important factor to consider in paediatric CD since treatment options are limited, and being able to maintain one strategy over time can be notably helpful. Notably, these patients received concomitant treatment with azathioprine, which probably contributed to the observed outcomes.

In group B, all patients achieved clinical remission on week six and maintained it on week 12, but only 60% maintained it by week 24. Although these results seem less impressive, it is encouraging to have a new option of treatment that has excellent efficacy in achieving clinical remission in the short term (12 weeks) and maintaining it in more than half of the patients. Sustained clinical remission on week 24 is similar to that described in previous literature (60%) [13]. FC and PCDAI did not improve significantly in group B. This lack of improvement can have a negative impact on patient outcomes perspectives. Still, it is unclear if these results are due to a lack of mucosal healing or a small sample size unable to reach statistical significance. Further studies are needed to evaluate the actual effect of CDED plus PEN in the biological improvement and mucosal healing in patients with loss of response to biologics. When we consider the complexity of this specific group of patients whose disease activity is higher at baseline, have a secondary loss of response to biologics and have fewer treatment options available, CDED plus PEN results in a promising possibility. Adherence and safety were remarkable in both groups and similar to those described in previous literature [22].

This study aimed to share our experience using CDED plus PEN to provide objective outcomes that could provide insight for clinicians currently using or intending to use diet in paediatric CD management. Nevertheless, we acknowledge that the study has limitations, including the fact that it is a retrospective observational study, which can limit the accuracy and completeness of the analysed data. The studied cohort is small and based on patients of a single care centre. We did not compare results with a control group using current gold treatment standards, EEN, but compared it to that published in previous literature [7, 18]. Another limitation is the possible bias in the evaluation of adherence as we did not have standardised tools to evaluate adherence and had to rely on notes in the clinical history. Finally, we were not able to include endoscopic evaluations of response throughout the follow-up in all patients. It would have been interesting to include endoscopic response to corroborate CDED plus PEN’s efficacy in endoscopic remission and to compare it with other studies [29].

## Conclusion

Treatment with CDED plus PEN was well tolerated, and patients had excellent adherence. Our cohort showed outstanding results at disease onset and promising results after losing response to biologics. CDED plus PEN is a new promising rescue strategy to treat CD after the failure of previous biologics safely and without needing to change the therapeutic target.

## Data Availability

All data generated or analysed during this study are included in this published article.

## List of abbreviations

CD: Crohn’s disease
CDED: Crohn’s disease exclusion diet
CRP: C-reactive protein
ENN: Exclusive enteral nutrition
ESR: Erythrocyte sedimentation rate
FC: Faecal calprotectin
PCDAI: Paediatric Crohn’s Disease Activity Index
PEN: Partial enteral nutrition
